# Injury patients’ perceptions of drink-driving: A qualitative assessment of drink-driving behavior in Moshi, Tanzania

**DOI:** 10.1371/journal.pone.0230662

**Published:** 2020-05-05

**Authors:** Deena El-Gabri, Loren K. Barcenas, Brian Meier, Mark Mvungi, Michael Haglund, Charles J. Gerardo, Joao Ricardo N. Vissoci, Catherine A. Staton

**Affiliations:** 1 Duke Global Health Institute, Duke University, Durham, NC, United States of America; 2 Division of Emergency Medicine, Department of Surgery, Duke University School of Medicine, Durham, NC, United States of America; 3 Kilimanjaro Christian Medical Center, Moshi, Tanzania; 4 Division of Neurosurgery and Neurology, Department of Neurosurgery, Duke University School of Medicine, Durham, NC, United States of America; University of California San Diego School of Medicine, UNITED STATES

## Abstract

**Background:**

Globally, about 2.3 billion people are current alcohol drinkers, and 283 million have an alcohol use disorder. Alcohol use while driving is a major contributor to road traffic injuries (RTI). We need to understand the culture and perception of drink-driving in this setting to understand why people continue to drink drive and allow policymakers to develop more effective ways to address drink-driving behavior. This study aims to qualitatively determine what injury patients, their families, and community advisory board members in Tanzania believe about drink-driving to help inform policies to address this problem.

**Methods:**

The semi-structured focus group was designed based on the grounded theory and assessed using thematic analysis. Focus groups participants were a convenience sample of injury patients, their families, and community advisory board (CAB) members. Analysis was iterative throughout the study. All transcripts were coded using a thematic narrative approach. Representative quotes for each theme were then selected based on comparative analysis of coding with input from research team members.

**Results:**

A total of ten focus groups were conducted (4 patient, 4 family, and 2 CAB) with a total of 104 participants (37 females and 67 males). The normalization of drinking among drivers has allowed this behavior to become ingrained in the culture. Participants expressed notions that passengers are responsible for their own safety, rather than drivers being responsible for their passengers. Most participants believe it is a citizen’s duty to inform the police of suspected drink-driving, however there were differing opinions about how effective informed police officers can be in practice. Focus group discussions between all three population types highlighted major themes of ‘drinking is ingrained in boda boda driver culture’, ‘individuals have a personal responsibility to address drink-driving’, and a ‘police enforcement on drink-driving is necessary’.

**Conclusions:**

Normalization of drink-driving in commercial driver culture creates a dangerous environment for passengers which can be mitigated by education and health promotion. As most passengers already take personal responsibility for their own road traffic safety, they may be likely to make use of safe ride options, if available. While legislation is in place against drink-driving, police officers need to be empowered with appropriate training and funding to enforce them.

## Introduction

Globally, about 2.3 billion people are current alcohol drinkers, and 283 million have an alcohol use disorder [1]. Alcohol use is the fifth leading risk factor for all disease and injury worldwide [2]. Road traffic injuries (RTIs) alone accounted for the most alcohol-attributable deaths in 2016 [1]. Those who drink while driving have 3.8 times greater risk of injury and fatality [3].

RTIs are particularly a problem in low- and middle-income countries (LMICs) with poor infrastructure and weakly enforced traffic laws [1,4]. In particular, laws related to alcohol use while driving must be firmly enforced in order to be effective at reducing alcohol-attributable RTIs [5,6]. High rates of alcohol attributable RTIs in many countries indicate that they either do not have proper policies in place, or they are not being implemented and enforced strongly enough [1]. With RTIs contributing to around 70% of all alcohol-related injuries, Tanzania falls into the second category [7]. Current policies in place include blood alcohol concentration limits of 0.08g/dl for the general population and 0g/dl for commercial drivers, which can be enforced through random breath testing or police check points [8]. However, the effectiveness of overall enforcement of national drink-driving laws is rated a 2 on a scale of 0–10 [8].

A total of 36 traffic crash hotspots have been identified in Moshi, Tanzania alone, with 40% of crash sites on local roads with increased motorcycle density [9]. One cross-sectional study done country-wide found that motorcycles caused 53.4% of road traffic crashes. The dangerous nature of motorcycles is troubling as many Tanzanians ride as passengers on motorcycle-taxis (called “boda bodas”) as a form of transport [10]. Another vehicle being used to meet the rising demand of public transportation in northern Tanzania is the minibus, locally known as dala-dala. Dala-dala drivers are incentivized to engage in unsafe driving habits such as ignoring road signs and speeding since they are paid on commission and do not depart until the minibus is full [11]. Studying RTIs in Tanzania is important to gain first-hand understanding of drink-driving behavior and ultimately reduce the burden.

Driver drunkenness is perceived as one of the leading causes of RTI in Tanzania [12], however not much is known about why people continue drink-driving regardless of the consequences, or what patients believe can prevent injuries like their own. Knowing the injury population’s views will allow policymakers to develop more effective ways to address drink-driving behavior, based on those who have experienced the consequences of drink-driving. The lack of cultural understanding makes grounded theory useful to investigate inductively [13]. This study aims to qualitatively determine what the injury population, their families, and community advisory board members of Tanzania believe about drink-driving to help inform policies to address this public health hazard.

## Methods

### Institutional review board

For the study, we obtained IRB approval from the Duke Institutional Review Board, Kilimanjaro Christian Medical Center Ethics Committee and National Institute of Medical Research. There was a minimal level of risk for this study. Patients completed a written informed consent prior to participation but to ensure privacy, names or identifying information of participants were not collected.

### Study design

The semi-structured focus group was designed based on the grounded theory and assessed using thematic analysis. Grounded theory is a research method that generates theories grounded in data [14]. Constant comparative analysis, a process in grounded theory used for category development [15], led to focus group questions (appendix A) about community drinking behavior and what is appropriate and acceptable behavior when deciding to or seeing someone drink and drive. Focus groups were conducted between August 2016 and January 2017.

Focus groups were conducted by two trained female research nurses at Kilimanjaro Christian Medical Center (KCMC). The research nurses both have over ten years of experience conducting focus groups among similar patient populations. A relationship was established with the participants at the onset of the focus group sessions. Focus group participants were informed of the aims of the study as well as the qualifications of the research team and focus group facilitators. They were informed that the focus group is a component of a greater research collaboration aimed at reducing alcohol related injury in northern Tanzania. Facilitators reported their interest in the research as based on an investment in the livelihood and betterment of the community.

### Study setting

This project took place in Moshi, a city in the Kilimanjaro region of Northern Tanzania with a population of 143,799. Moshi is home to Kilimanjaro Christian Medical Center (KCMC), the third largest hospital in the country and the referral hospital for northwestern Tanzania [16]. KCMC serves the urban and rural population of Moshi and was therefore selected as a central location to assess general perceptions of the region. Assessing the qualities and factors that lead to drink-driving is important for this region as road traffic crash rates are climbing and current data from the KCMC Casualty (Emergency) Department suggests that 28% of all the patients who arrive to the Casualty Department for treatment of an injury consumed alcohol prior to their injury and therefore are ‘Hazardous drinkers’ [17].

Although Tanzania does have a national blood alcohol limit of 0.08g/dl for the general population and 0g/dl for commercial drivers [8], policies including random breath testing, police checkpoints, and penalty point systems are currently not in place [8]. High alcohol consumption is more of a problem in Moshi than other regions of Tanzania because it is less religious than other Muslim predominated regions [18]. The strong Chagga culture of the region views alcohol as an integral part of traditional ceremonies, thus making it a more acceptable habit [19]. Another factor contributing to higher alcohol consumption is the large tourist population attracted by being situated at the base of Mount Kilimanjaro. Tourism brings in significant revenue that contributes to higher disposable income, which is associated with higher alcohol consumption rates [18]. Demand for bars created by tourists could contribute to the wide availability and exposure to advertisements of alcohol experienced by northern Tanzanians [18].

### Study population

Focus groups participants were a convenience sample of injury patients, their families, and community advisory board members. Patients and their family members were identified face-to-face in the KCMC ED waiting or treatment areas, after treatment or stabilization, and were offered participation in the focus group. If patients or family members accepted, they were invited to return to the hospital for patient and family focus group days, respectively.

Injury patients (both patients and drivers) were included in the focus groups if they were ≥18 years of age, seeking care at KCMC for any acute (<6 hours) injury, clinically sober at the time of enrollment, medically stable, able to communicate in Swahili or English and consent to participate. Patients were excluded from enrollment if they were medically unstable or had a deteriorating condition, were too critically ill to participate, did not speak English or Swahili, were <18 years of age, presented for non-injury related complaints or follow-up care, or did not consent to be enrolled. Family member focus group participants were family members of a patient who was able to be enrolled in the study, who also agreed to participate, and speak English or Swahili.

Research nurse facilitators attended Community Advisory Board (CAB) meeting to conduct focus groups in order to utilize this group's perspective on drinking behavior within the community. The KCMC CAB is comprised of 30–40 adult community members who understand research, have advised investigators on pertinent research questions, cultural norms, and cultural acceptability of interventions, treatments and research protocols. All interested CAB participants enrolled were ≥18 years of age and were present at the monthly CAB meeting when we conducted the focus group discussion.

### Data collection

Patient and family focus groups were scheduled once 5–10 eligible interested participants were recruited from the emergency department and provided informed consent. CAB focus groups took place prior to the monthly CAB meeting and consisted of 5 to 10 members who provided informed consent. No participants dropped out. Each focus group, led by trained research nurses, lasted between 45 and 60 minutes. They were audiotaped and transcribed for formal qualitative analysis utilizing thematic analyses. Transcriptions occurred within days following the focus group and research nurse notes were included in the transcriptions about the content.

Focus groups among each population group, patients, families, and CAB members, were conducted using an iterative process until thematic saturation was reached. This was done for four patient, four family member, and two CAB focus groups. The original focus group questions were designed to understand the general perceptions of drink-driving in Moshi Tanzania. These questions included “What is acceptable drinking behavior?” and “What is thought of a person who drinks and drives?”. These scripted questions were piloted by the research nurses conducting focus groups. After thematic saturation was reached, the focus group script was altered to expose reasons for preliminary data from patient surveys. Focus groups were conducted until thematic saturation was again reached.

After the focus groups were recorded and transcribed, each script was translated from Swahili to English. English scripts were then assessed for potential cultural misinterpretations, back translated and annotated for English, American comprehension. Transcripts were assessed by bilingual American and Tanzanian researchers and not returned to participants as they were difficult to contact after completion of the study. All transcripts, audio tapes, and related data will be kept for six years after study completion.

### Data analysis

Analysis was iterative throughout the study, which allowed emerging themes, derived from the data to be explored in later focus groups. The focus groups among patients, family members, and CAB members were coded separately and then analyzed. Comparing and contrasting across and within these datasets highlighted emerging themes and divergence of perspectives. Thematic saturation occurred when no new themes developed from focus group analysis and marked the end of the qualitative study for individual population subsets.

All transcripts were coded by DE and BM, using a thematic narrative approach, reflecting the research questions and themes raised by the participants [20]. The researchers (DE and BM) separately completed coding with primary and secondary level coding classifications. DE and BM then compared coding with advisors specializing in qualitative research. The Tanzanian research group reviewed the evolving thematic codes and resulting narratives and gave input based on their experience with the focus group populations and cultural knowledge. Representative quotes for each theme were then selected based on comparative analysis of DE and BM coding with input from research team members.

## Results

### Demographics and characteristics of participants

A total of ten focus groups were conducted (4 patient, 4 family, and 2 CAB) with a total of 104 participants (37 females and 67 males). Demographic information is not presented here in the interest of maintaining respondent confidentiality. While the study was not intended to focus on commercial drivers, most participants spoke about boda boda or dala dala drivers. Focus group discussions between all three population types highlighted major themes of ‘drinking is ingrained in boda boda driver culture’, ‘individuals have a personal responsibility to address drink-driving’, and a ‘police enforcement on drink-driving is necessary’.

### Emerging themes and related quotes

#### 1. ‘Drinking is ingrained in boda boda driver culture’ theme

Among all three focus group types, a prominent theme was that drinking is so common among boda boda drivers that it is seen as normal. The normalization of drinking among boda boda drivers has allowed this behavior to become ingrained in the culture, so *“reporting those drivers is very rare”* (Family, 4) making it difficult to change. Along with the perception that drink-driving is normal comes the normalization of its consequences. One focus group member admits that, *“Although accidents still happen and people cut their leg or break their head [due to alcohol attributable road traffic injuries]*, *our society sees it as normal”* (Family, 4).

A strong drinking culture may stem from occupational stressors. Boda boda driving is seen as a difficult job and therefore deserving of a drink. Society believes that alcohol gives drinkers more energy, so *“boda boda drivers cannot work well until they drink something”* (Family, 4). Additionally, drivers believe that because they are both experienced drinkers and drivers that they can do both successfully. One patient related that *“I am experienced with alcohol*. *I can drink even four containers so that I have energy to work”* (Patient, 4).

Drinking is so ingrained in the boda boda driver culture that fellow drivers have been known to *“help one another buy alcohol by contributing money if one person cannot afford a drink”* (Patient, 1). This implies that boda boda drivers find camaraderie through drinking, making it even more difficult to stop. Reverse stigma, or a negative attitude among heavy drinkers towards someone who stops drinking, may play a role in perpetuating drink-driving culture among boda boda drivers. For example, if one driver quit drinking, *“his fellows will see him as a betrayer*. *They will want to pull him back to his previous ways so he cannot escape their company of drinking alcohol”* (CAB, 1).

#### 2. ‘Individuals have a personal responsibility to address drink-driving’ theme

Throughout focus groups, participants expressed notions that passengers are responsible for their own safety, rather than drivers being responsible for their passengers. Generally, there were two responses showing different levels of commitment to personal safety. Some act passively, not taking any action against drunk driving because they feel that drink-driving is inevitable and changing this behavior is hopeless. Others look out for the personal safety of themselves and others by taking actions such as looking for a sober driver and stopping a drink driver.

Participants who act passively typically know the dangers of drink-driving, but feel that trying to change this behavior is useless. Because they feel that drink drivers *“won’t listen to anyone or ask for any assistance”* (Patient, 2) these participants reluctantly accept drink-driving. Most passengers maintain the idea that those drivers who drink are set in their ways because past confrontations with drivers have not made a difference and have become passive towards drink-driving as a result. One person from the CAB focus group recalls trying to positively influence a drink driver, but was unsuccessful, saying, *“We have tried to advise him but he said he can’t stop drinking alcohol until he dies*.*”* The perception that changing drink-driving behavior is impossible discourages citizens from taking action or pushing for stronger law enforcement.

Rather than feeling hopeless and becoming passive, another sub-group of participants took personal safety into their own hands. One way of doing so is refusing to ride with a drink driver and finding another driver. Participants said, *“If I find a dala-dala driver is drunk*, *I must go find another dala-dala driver”* (Family, 2) and *“It’s better not to enter the bus if you realize that the driver is drunk”* (CAB, 2). Participants in this group believed that safety was important enough to intervene, using methods to stop drink drivers. If the passengers have not gotten into the vehicle yet, they will avoid riding with the driver and *“tell fellow passengers that the driver is drunk”* (CAB, 2) while *“disagreeing [that the driver is ready] because he is drunk and then report him”* (CAB, 2). Alternatively, “*If there is only one driver on whom we depend and he is drunk*, *he will have to rest until the drunkenness is over*. *Then we will ask him to drive” (CAB 2)*. If the passengers are already in the vehicle, and see that *“he is losing concentration*, *then we use force to stop him and tell him not to drive the vehicle”* (Patient, 3). These passengers understand the danger of riding with a drink driver and believe taking responsibility for their own safety is necessary.

#### 3. ‘Police enforcement on drink-driving is necessary’ theme

The final contributing theme that emerged from the perceptions of drink-driving focus groups was ‘necessary police enforcement on drink-driving’. Most participants believe it is a citizen’s duty to inform the police of suspected drink-driving, however there were differing opinions about how effective informed police officers can be in practice.

Those who thought citizens should be actively involved expressed that *“passengers should be very careful with a drunk driver and inform the traffic police immediately”* (Patient, 2). Reflecting the thought that police should be informed, one participant notes, *“If you know he is drunk then you have to stop him driving a car or inform a policeman that he is drunk and can’t drive the vehicle”* (Family, 2).

In practice, informing the police should be simple, *“it’s just a matter of reporting the driver who is drunk*. *He will be stopped and you will be given another driver*, *therefore it’s very easy”* (CAB, 2).

However, others feel that even if you do go to the law enforcement, not much can be done to resolve the situation. In reply to the previous quote, a CAB member explained that in his or her experience, getting a sober replacement driver can be difficult and very slow, pointing to the need for improved policies and police enforcement. Another issue is the reliability of the passengers’ report in the police’s eyes. If a law enforcement officer cannot confirm that a driver is drunk, he or she may not take any precautions, as this participant describes, *“If three people from the same bus tell [the police] that the driver is drunk*, *he may not believe them*. *It would take the bus getting into an accident for him to believe”* (CAB, 2). Because the police do not take the people’s word, many feel that the police are not doing enough to enforce drink-driving laws.

## Discussion

This innovative study is the first to examine the perceptions of drink-driving in the Moshi, Tanzania community as experienced by injury patients, their family members and a community advisory board. Focus group discussions demonstrated three specific themes: 1) that drinking is ingrained in boda boda driver culture, 2) if individuals take responsibility to address drink-driving it is at the personal level, and 3) police enforcement needs to be strengthened. These results highlight areas that potential interventions could focus on to be the most productive; For example, the normalization of drink-driving among boda boda drivers needs to be addressed, utilizing personal motivation to avoid drink-driving exposure could be increasing safe ride options, and there is a perception that strengthening enforcement would decrease drink-driving.

We found that drink-driving behavior is specifically ingrained in boda boda driver culture. While previous studies have shown that drinking alcohol is a regular part of social interactions throughout Tanzania, [21,22] we found that it was even more common place and expected part of this work culture. Some commercial drivers in Tanzania see alcohol as a means of fighting fatigue [21], and when workers perceive alcohol to enhance their performance or reduce stress, it is more likely to become widely accepted [21,22]. Dominant cultural practices can be addressed by health promotion initiatives at the community level. For example, the huge reduction in tobacco use that followed a cultural change from tobacco accomodation to intolerance is a great illustration of the effectiveness of health promotion initiatives [23]. Interventions targeted at communities shown to reduce alcohol use include group-based education programs, changing school policies, and emphasizing alcohol-related harms [24].

We also found that if individuals take responsibility to address drink-driving, it is at the personal level. We can leverage the personal responsibility most passengers feel for their own safety by providing more alternatives to drink-driving or riding with impaired drivers. Safe ride programs are the provision of free to low cost transportation, which have been implemented differently in various high-income settings with mixed effectiveness [25]. The United States National Highway Traffic Safety Administration reports that programs with the greatest likelihood of reducing crashes incorporate accessibility, availability, and ease of integration into activity [26]. However, these programs may function differently within Tanzania where most of our participants discussed riding with a commercial driver or taking public transport. Therefore, these programs would need to be evaluated to see if both intoxicated and sober individuals would choose to use a safe ride program than ride with an intoxicated driver, among other feasibility and acceptability challenges. An alternative transportation option is using ridesharing apps, such as Uber and Lyft [27,28]. Ridesharing apps allow passengers to order a safe ride from their phone at any time, as long as a driver is available. To remain a certified driver with the company, drivers would have to comply with the company’s zero tolerance drug and alcohol policy, and ride users can leave evaluations of the drivers which would reduce the use of those drivers with poor evaluations [29,30].

When taking into context the overwhelming cultural acceptance and the fact that many take personal responsibility for self-protection, one intervention that can address education and harness personal choice would be a brief intervention (BI). The BI has been used in emergency departments in high income countries to encourage the reduction of alcohol consumption among injury patients [31,32]. While there is no evidence to support its use in countering cultural practices on a large scale, patient level education can increase personal choice and capacity for change. Focusing on topics important to the individual, including perceived risks, moral convictions, or religious beliefs can help curb alcohol consumption [25–27]. Using the BI in medical facilities is highly effective at reducing self-reported drinking as well as alcohol-related road traffic injuries [25]. However, these interventions must be tested and adapted for culturally appropriate use in Tanzanian society.

Participants identified a perceived lack of police enforcement of legislation against drink-driving. Proper police enforcement of traffic laws is important as it is associated with decreased road traffic fatalities and injuries [16]. Using breathalyzers and police checkpoints to apprehend drink drivers can give officers agency to enforce consequences that impact behavior [33,34]. However, police officers in Tanzania are not empowered to enforce current legislation due to constraints on funding, human resources, equipment, and vehicles [35]. Additional barriers faced by the Tanzanian police force include inadequate training in road safety over the last few years, as well as lack of motivation in part due to criticisms of abuse of power [35]. A multi-level intervention focusing on improving policy, supporting education and availability of safe rides, as well as supportive police enforcement would be successful [16].

### Limitations

One limitation of the study is the potential for social desirability bias, especially on a stigmatized topic such as drink-driving. To limit the effect of this potential bias, we used research personnel who were comfortable talking about stigmatizing topics and included questions to talk about the general society and not the participants individual actions or behaviors. As inclusion criteria required participants to be injury patients who were medically stable to enroll in the study, we lack the perspective of those who were critically ill or in deteriorating condition. The fact that we enrolled injury patients and their family allows us to understand the motivations for continued drink-driving to plan for interventions, but might not fully represent the Tanzanian general society’s perspective about drinking or drink-driving.

## Conclusions

Normalization of drink-driving in commercial driver culture creates a dangerous environment for passengers. Interventions like education and health promotion can help mitigate drinking and spark change among commercial drivers. As most passengers already take personal responsibility for their own road traffic safety, they may be likely to make use of safe ride options, if available. Implementing the BI in emergency departments can encourage high risk drinkers to take personal responsibility for their drinking behaviors as well. While legislation is in place against drink-driving, police officers need to be empowered to enforce them. With training and funding, police officers can implement checkpoints for random breathalyzer tests, which has been shown to decrease drink-driving and save lives.
